# An Examination of the Relationship Between Social Support Networks and Opioid Misuse Among American Indian/Alaska Native Populations: A Systematic Review

**DOI:** 10.3390/healthcare13162072

**Published:** 2025-08-21

**Authors:** Samuel Asante, Allen Shamow, Eun-Jun Bang

**Affiliations:** School of Social Work, Northeastern State University, 3100 E New Orleans St., Broken Arrow, OK 74014, USA; shamow@nsuok.edu (A.S.); bang@nsuok.edu (E.-J.B.)

**Keywords:** social network, social support, risk factors, protective factors, opioid misuse, American Indian, Alaska native

## Abstract

**Background/Objectives**: This systematic review addresses the disproportionate impact of the opioid epidemic on American Indian and Alaska Native (AI/AN) populations by examining the socio-ecological and social network factors that influence opioid use and misuse. While previous reviews have largely focused on treatment modalities or structural determinants such as socioeconomic status and rurality, few studies have explored the role of social networks as risk or protective factors, particularly within AI/AN communities. **Methods**: Applying the Preferred Reporting Items for Systematic Reviews and Meta-Analyses (PRISMA) framework, the review synthesized findings from three scholarly databases (PubMed, EBSCOhost, ProQuest), six institutional repositories (e.g., Indigenous Studies Portal), and one academic search engine (Google Scholar). Studies that examined the influence of social network domains on opioid misuse in AI/AN populations in the United States, reported quantitative or qualitative data, and were published between 2010 and 2022 were included in this review. Study quality was assessed with the JBI Checklists for Analytical Cross Sectional Studies and Qualitative Research. Of the 817 articles initially identified, 7 met the inclusion criteria, with most studies focusing on AI/AN adolescents and young adults, a demographic shown to be especially susceptible to opioid misuse. **Results**: The review identified several social network domains that significantly affect opioid use patterns, including familial relationships, peer associations, community dynamics, educational influences, cultural traditions, social media engagement and the effect of historical and intergenerational trauma. These domains can function either as protective buffers or as contributing factors to opioid misuse. **Conclusions**: The findings underscore the necessity for future longitudinal research to elucidate the causal pathways between these social network factors and opioid behaviors, particularly concerning trauma and digital media exposure. Furthermore, the study highlights the importance of culturally grounded, evidence-based prevention strategies that address the multifaceted social environments of AI/AN individuals. Such approaches are critical to fostering resilience and mitigating the opioid crisis within these historically marginalized populations.

## 1. Introduction

Opioid usage continues to be a key public health concern within the United States. Despite increased federal funding to address the growing opioid problem [1], there have been over 500,000 opioid-involved deaths in the United States since the year 2000 [2]. Of the more than 67,000 drug overdose deaths that occurred within the United States in 2018, nearly 70% dealt with either prescription or prohibited opioids [3]. Not only does the United States have the highest number of deaths due to opioids per capita, but this number exceeds the median for countries that are part of the Organization for Economic Cooperation and Development by five [4]. While the deleterious effects of the opioid crisis are evident across various segments of the U.S. population, the American Indian/Alaska Native (AI/AN) population in particular has dealt with the harmful consequences of this growing issue.

An increase in health challenges and higher rates of substance use disorders (SUDs) among AI/AN people have been linked to the historical trauma they experienced related to their forced removal and loss of land along with the efforts aimed at their forced assimilation [5,6]. In terms of SUDs, AI/AN people have the highest frequency of alcohol and tobacco use disorders but also account for incidence and mortality rates associated with opioid usage that are higher than the general population [7]. Between 2000 and 2019, the opioid-related overdose death rate among AI/AN populations saw a drastic increase from 2.7 per 100,000 in 2000 to 17.0 per 100,000 in 2019 with the rate in 2019 eclipsing the national rate of 15.2 per 100,000 [2]. A jarring statistic is the fact that AI/AN people account for less than two percent of the total U.S. population [8] yet had an opioid-related death rate that surpassed that of White Americans in 2020 [9]. These statistics illustrating the pervasiveness of the opioid crisis among AI/AN populations magnify the need to identify the factors serving both to inhibit and facilitate the problematic effects of opioids among this group.

Terms such as social support and social networks are often strewn about synonymously. Heaney and Israel [10] explain the distinction between these concepts with social networks pertaining to existing social relationships and social support, which is variable in nature, referring to an intended task of these relationships. Among AI/AN youth, sources of social network support that may impact their mental health include their connection to their culture, the nature of their tribal and spiritual ties, and their relationships with family and peers [11,12]. Proper behavioral health maintenance is particularly critical given the link between poor mental health and increased SUD risk [13], with higher levels of loneliness being connected to an increased likelihood of opioid misuse [14]. This interplay between support, mental health, and SUDs underscores the need for a comprehensive evaluation of the sources of social network support among AI/AN populations given their experience with the opioid crisis.

Many prior systematic reviews assessing the challenges of opioid usage have focused on the treatment aspect [8,15,16], while other systematic reviews have focused on the impact of factors such as socioeconomic marginalization and rural conditions on opioid-associated harm and death [17,18]. Cance et al. [19] conducted a systematic review evaluating the effect of social connection, a concept that often subsumes concepts such as social support and social networks, on opioid misuse and opioid use disorder (OUD). Yet the systematic review by Cance et al. [19] does not focus on any specific population, let alone a population that has dealt with the challenges of the opioid crisis such as AI/AN populations. Further support for examining the relationship between social network support and opioid misuse among AI/AN populations stems from the fact that prior research has identified a link between opioid misuse and increased suicide attempts among AI/AN youth [20]. An additional consideration and potentially distinguishing characteristic of the present study is that social networks such as the family cannot be characterized by traditional conceptualizations due to the strong ties among extended family members and those not related by blood among AI/AN populations [21]. An opportunity to take stock of the research involved with the relationship between social network processes and opioids among AI/AN populations will not only expand the current knowledge base but inform interventions focused on ameliorating conditions among this group. It is for these reasons that this systematic review assesses the relationship between social network support and opioid misuse or opioid use disorder (OUD) among American Indian and Alaska Native (AI/AN) populations in both quantitative and qualitative studies in the United States since 2010. The review was structured to address the following research question:

- What are the domains of social network that serve as protective and/or risk factors of opioid misuse or opioid use disorder (OUD) among American Indian/Alaska Native (AI/AN) populations?

## 2. Methods

A systematic review of the published literature was conducted in accordance with the Preferred Reporting Items for Systematic Review and Meta-Analyses (PRISMA) guidelines [22] to identify social network support sources that may act as protective or risk factors for opioid use or misuse in American Indian/Alaska Native (AI/AN) communities. The study was registered with the Open Science Framework systematic review registry (registration DOI: https://doi.org/10.17605/OSF.IO/S29DE), accessed on 1 August 2025.

### 2.1. Initial Search

Literature searches of ProQuest, PubMed, EBSCOhost (academic search elite, health source nursing, APA PsychArticle, APA PsycInfo, Social Science full text, Eric, Academic Search Premier), Google Scholar, and institutional repositories including the Indigenous Studies Portal (I-Portal) of the University of Saskatchewan and the scholar(ly) commons of the University of North Dakota, University of Utah, University of Oklahoma, Arizona State University, and University of South Carolina were conducted to identify studies published on the relationship between social support networks and opioid use or misuse among American Indian and Alaska Native populations based on the search terms provided in Table 1. Given the potential for COVID-19-related factors to significantly alter opioid use and misuse patterns, the search for data was intentionally restricted to studies published between 2010 and 2022. All three researchers of this study were involved in the data search and retrieval process.

**Figure 1:**
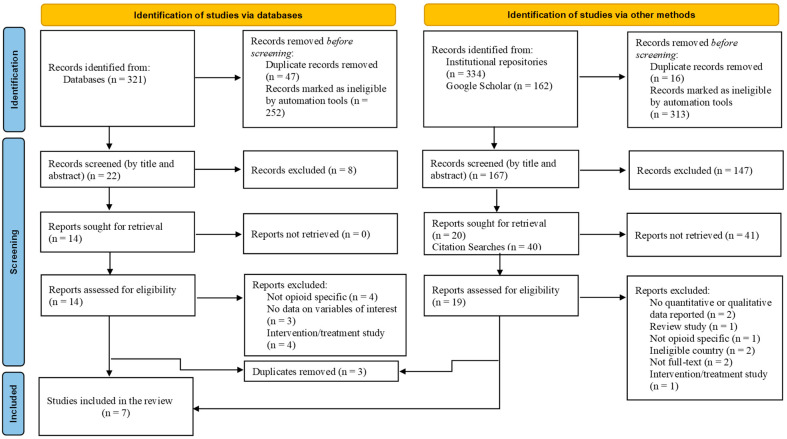
PRISMA flow diagram.

The searches identified 817 studies, published between 2010 and 2022. The numbers of studies retrieved from databases and institutional repositories were as follows: ProQuest (*n* = 247), PubMed (*n* = 58), EBSCOhost databases (*n* = 16), Google Scholar (*n* = 162), Indigenous Study Portal (I-Portal) (*n* = 309), and scholar(ly) commons of the University of North Dakota (*n* = 6), Arizona State University (*n* = 3), University of Utah (*n* = 6), University of Oklahoma (*n* = 3), and University of South Carolina (*n* = 7). Book chapters, dissertations, study reviews, news items, and magazines were excluded from the total number of studies obtained in order to maintain a more standardized quality level. Additionally, publications that examined the issue being considered in this study in populations other than indigenous populations and studies published outside the United States were also excluded (see Table 2). These steps removed 565 studies from the total studies obtained in the initial search. Of the remaining 252 studies, 63 were exact duplicates and were excluded. This produced a total of 189 studies that were screened for inclusion, as shown in Figure 1 on the PRISMA diagram chart.

### 2.2. Screening Articles for Inclusion

The 189 studies were screened by their titles and abstracts (as shown in Figure 1). Studies were retained if they examined social networks (e.g., family, peer, community) and opioid use or misuse among American Indians and Alaska Natives, quantitatively or qualitatively assessed social networks and opioid use or misuse, and reported results in peer-reviewed articles published in English. Given the limited number of studies published on this topic, it was prudent to include qualitative studies along with quantitative studies in the review. This step produced a total of 34 studies, which were sought for retrieval. Reference and citation searches were conducted on all relevant studies. This produced 40 studies for screening, of which 3 were duplicates, and the remaining 37 were deemed ineligible. Of the 34 studies sought for retrieval, 1 was found to have been published in an ineligible country. The remaining 33 studies were assessed for inclusion in the review. Most studies were excluded on the grounds of study type: intervention studies and study protocols on opioid use in indigenous populations. Other studies were excluded on the basis of the study population: indigenous populations living outside the United States. Some were also excluded on the basis of type of substance examined: alcohol and tobacco (not opioid-specific studies). Studies which had no full text available and those that reported no qualitative or quantitative data were excluded. A total of seven studies met the inclusion criteria and were included in this review.

### 2.3. Quality Assessment Tool and Data Extraction Process

The article screening and eligibility process was completed independently by two of the researchers of this study. An unstandardized protocol with clear criteria for screening was developed and used to minimize discrepancies in screening outcomes and enhance the validity of the screening process. Articles were selected only if both reviewers agreed on the articles’ quality, assessed with the Joanna Briggs Institute (JBI) Checklist for Analytical Cross Sectional Studies [23] and the JBI Critical Appraisal Checklist for Qualitative Research [24]. The JBI Checklist for Analytical Cross Sectional Studies is used to assess the methodological quality of cross-sectional studies in systematic reviews. The checklist has 8 items, detailing criteria for inclusion, validity, reliability, and the identification of confounding variables that could impact a study’s results and interpretation of findings [23]. The JBI Checklist for Qualitative Research is a tool used to assess methodological quality and congruity of qualitative studies in systematic reviews. The checklist covers 10 criteria, assessing, for instance, how a study’s methodology aligns with its philosophical perspective, research questions and objectives, data collection and analysis methods, the interpretation of results, conclusions drawn based on the analysis or interpretation of data, and the researcher’s role in the qualitative research process [24].

Both JBI checklists serve as valuable tools for researchers, as they offer quick and efficient ways to evaluate the methodology of studies and improve the synthesis of evidence [23,24]. Any disagreement between the reviewers was addressed by the third researcher. The Cohen Kappa (agreement) between the two reviewers was 0.76 for the quantitative studies and 0.74 for the qualitative studies, indicating substantial agreement [25]. Following the screening, data from each article (pertaining to author, year, study title, objective, design, type of opioid, social network domains, statistical analysis tool, results, and conclusion) were extracted onto an Excel spreadsheet.

### 2.4. Rationale for Omission of Formal Risk-of-Bias Assessment

This systematic review focused on non-intervention studies—primarily qualitative, descriptive, and observational research—examining the relationship between social support networks and opioid misuse among American Indian and Alaska Native (AI/AN) populations. Because the included studies did not involve randomized controlled trials or other experimental designs, conventional risk-of-bias tools (e.g., Cochrane RoB 2 or ROBINS-I) were not appropriate or applicable. These tools are designed to assess internal validity in intervention studies and rely on criteria such as allocation concealment, blinding, and intervention fidelity, which are not relevant to the study designs included in this review. Instead, the study prioritized the contextual richness and methodological transparency of the studies included in the review. This allowed for an evaluation grounded in the nuances of community-based and culturally embedded research involving AI/AN populations, where rigid standardization may be neither feasible nor appropriate.

## 3. Results

### 3.1. Methodological Characteristics of Included Studies

Applying the Preferred Reporting Items for Systematic Reviews and Meta-Analyses (PRISMA) framework, the review synthesized findings of studies published between 2010 and 2022 on social support network sources that serve as protective or risk factors for opioid use or misuse among AI/AN populations. The initial search identified 817 studies, of which 189 were screened by their titles and abstracts after records marked as ineligible by automation tools and duplicates were removed. After a thorough screening and eligibility assessment process, a total of seven studies met the inclusion criteria and were used in this review. Information on methodological characteristics and findings of the included studies is presented in Table 3 and Table 4, respectively.

#### Study Sample

The seven studies examined in this review include research published in seven academic journals. Five research teams that involved 48 different researchers authored all seven studies. Of the seven studies, five were quantitative and two were qualitative studies. All seven studies utilized a cross-sectional design. This limited number of both quantitative and qualitative studies, as well as the relatively small number of researchers focused in this area of scientific enquiry may increase potential bias in the evidence presented. The sample sizes of all studies varied greatly, ranging from as low as 21 to as high as 42,098, as presented in Table 3. The total number of participants included in all seven studies was 66,193 and consisted of students in grades 7–12 (*n* = 52,155), college students (*n* = 8094), youth in the community (*n* = 83), and healthcare professionals or providers (*n* = 21). Out of the 66,193 participants, 25,735 were self-identified AI/AN individuals.

### 3.2. Prevalence and Types of Opioid Use

The majority of the included studies utilized data from nationally recognized surveys or sources. These sources included the New Mexico Youth Risk and Resiliency Survey (NM-YRRS) [20,29], Our Youth, Our Future Survey (OYOF) [27], the American Drug and Alcohol Survey (ADAS) [15], and the American College Health Association National College Health Assessment II (ACHA-NCHA II). Data for the qualitative studies included in this review came from the California Tribal and Urban Opioid Needs Assessment [13,28], the largest publicly known participatory action research on opioid and substance use involving native populations. Five of the studies included in this review utilized school-based populations, with samples drawn from middle school to college. Participants for the remaining two studies came from community-based populations, including youth and healthcare professionals or service providers (see Table 3).

The type of opioid used among AI/AN populations was specified in six of the studies in this review. The types of opioids mentioned that were used included codeine, Vicodin, OxyContin, hydrocodone, Percocet, opium/opiates, Demerol, heroin, morphine, and methadone [13,14,15,20,27,28]. Although the Zeledon et al. [28] study did not have any active or past users in the sample, the healthcare professionals who participated in the study mentioned heroin as one of the opioids used by their service users. While the current research focused on opioid use among the AI/AN populations, some of the studies included in this review identified other commonly abused substances by study participants. Substances mentioned by studies included alcohol (3), cocaine (2), amphetamines/methamphetamine (3), cigarettes/tobacco (2), cannabis/marijuana (2), and benzodiazepines (1). One study used the term “other substances” without providing further specification (see Table 3).

For most of the included studies, opioid outcomes were measured as lifetime use/misuse and/or past-month (past-30-day) use (4) or use within the last 30 days or 12 months (1). Two studies did not use such measures. One study broadly asked whether participants have used any opioids or substances from a list of drugs provided, and the other had participants indicate common opioids and substances that impact AI/AN communities (see Table 3). Three of the included studies assessed whether opioids used by participants were prescribed or non-prescription use; two studies assessed the general use/misuse of opioids rather than categorizing them as prescribed or non-prescription use/misuse; one study assessed non-medical prescription opioid use among participants; and one study required participants to name opioids commonly seen among those dealing with substance use disorder in the Native American population.

One of the studies that examined the prevalence of opioid use in AI/AN populations is the study by Hirchak et al. [26] The study specifically examined lifetime non-medical prescription opioid use prevalence rates in 2013, 2015, and 2017 among urban and rural AI/AN and non-AI/AN middle- and high-school students. The study involved 42,098 participants, of whom 7037 were self-identified AI/AN. Although no specific percentages were reported, the rate of lifetime non-medical prescription opioid use in AI/AN students, as the study found, was lower compared to non-AI/AN students across all years.

Prince et al.’s [27] study utilized a machine learning application to determine risk and protective factors for past-30-day opioid use in a sample of 6482 middle- and high-school students, of whom 3098 self-identified as AI/AN. Although the study did not specify the percentage of AI/AN students who have used opioids within the past 30 days, the rate of opioid use in the total sample was three percent.

Nalven et al.’s [15] study explored the influence of family, school, and peer factors on opioid and heroin use in a sample of 3498 AI youth. The prevalence of past-month opioid misuse was 4.1%, lifetime opioid misuse was 14.7%, past-month heroin use was 1.1%, and lifetime heroin use was 2.8%. Qeadan et al.’s [14] study examined in a sample of 8094 AI/AN/NH (Native Hawaiian) college students whether problems with social bonds affect the misuse of opioids. The prevalence rate of opioid misuse in the sample was 7.2%. The rate of opioid misuse was higher among individuals who were aged 21–24 (7.68%), were transgender (17.27%), volunteered more than 40 h per week (30%), had D/F GPA (27.55%), felt unsafe on campus (15.38%), had financial problems (9.54%), felt lonely (9.00%), had difficulty in social relationships (10.48%), had family problems (9.26%), and experienced intimate partner violence (14.87%).

Agyemang et al.’s [20] study examined trends in opioid misuse, social support, and suicide attempts in a sample of 3641 AI/AN high-school students. The prevalence rate of opioid misuse in the sample was 12.9%. The rate was higher among individuals who reported low social support (18.9%), were female (13.0%), had poor grades (C, D, or Fs) (17.7%), lived on non-tribal lands (13.5%) and in rural areas (14.1%), those whose parents had less than high-school-level education (14.9%), and those who identified as gay/lesbian (32.5%).

West et al.’s [13] study examined AI/AN youth experiences with opioid and other substance use disorders in AI/AN communities in California. The sample involved 83 AI/AN youth. While no specific percentages were provided about the use or misuse of opioids in the sample, participants described opioids as being one of the most prevalent substances in their community. About 19% of the sample reported using marijuana at some point.

Zeledon et al.’s [28] study sought to identify facilitators and barriers to treatment of substance use disorders (SUDs) and opioid use disorders (OUDs). The study was conducted among 21 health professionals, of whom 14 self-identified as AI/AN. When asked to name substances that impact the AI/AN community, 43% reported opioids, 62% reported marijuana, 95% reported alcohol, and all mentioned methamphetamines. While the number reporting opioids does not represent a sample of individuals who have used or misused opioids at some point in their lives, it gives a general indication of the severity of the opioid problem among AI/AN populations.

### 3.3. Risk Factors of Opioid Use/Misuse

Most of the studies included in this review identified several social risk factors of opioid use among AI/AN populations (see Table 4). Notable among the factors were those that centered on human relationships. In Qeadan et al.’s [14] study, for instance, opioid misuse was highest among college students who reported experiencing loneliness, difficult social relationships, family problems, and intimate partner violence. Similarly, in Agyemang et al.’s [20] study, middle- and high-school students who reported receiving low social support from family, school, community, and peers were more likely to misuse opioids and had higher rates of suicide attempts than students who reported receiving higher levels of social support. Nalven et al. [15] also found that American Indian adolescents who reported lower family disapproval of substance use were more likely to have misused opioids during the past 30 days or report lifetime misuse of opioids (see Table 4).

In West et al. [13] and Zeledon et al.’s [28] studies, the two qualitative papers included in this review, participants pointed to family (e.g., “having problem at home”) and community stressors (e.g., violence and discrimination), family substance use, disconnection from community and culture, and, most importantly, historical and intergenerational (or multigenerational) trauma as factors that place AI/AN populations at significantly greater risk for opioid and substance use or misuse. Both West et al. [13] and Zeledon et al. [28] also identified normalization of substance use within families and communities and among peers as having the potential to put individuals at higher risk of opioid use or misuse (see Table 4).

Noting peer pressure as an important factor influencing opioid use, Prince et al.’s [27] study found that American Indian youth were more likely to use opioids if they had more friends pressuring them to use illicit drugs. Similarly, Nalven et al. [15] also found greater peer substance use was associated with greater likelihood of both lifetime and past-30-day opioid misuse among American Indian adolescents.

### 3.4. Protective Factors of Opioid Use/Misuse

Like the risk factors reported above, the studies included in this review identified several protective factors of opioid use among AI/AN populations. In their study examining trends in non-medical prescription opioid use among urban and rural middle- and high-school AI/AN and non-AI/AN students in New Mexico from 2013 to 2017, Hirchak et al. [26] found no significant differences in opioid use between AI/AN and non-AI/AN students. Results of their study, however, showed that family (e.g., having caring adults) and community support (e.g., community involvement), and clear rules at school, inhibited opioid misuse over the study period. Nalven et al. [15] found greater family disapproval of substance use was associated with lower odds of both past-month and lifetime opioid misuse. Prince et al. [27] also found in their study that having fewer friends pressuring you to use illicit drugs protected against recent (past-30-day) opioid use or misuse in AI youth. Snapchat use was also found to be a protective factor, with individuals using Snapchat often being less likely to report recent opioid use (see Table 4).

In their study, Qeadan et al. [14] specifically identified older age, higher GPA, and feeling safe on campus as strong protective factors for non-prescription opioid use. While their study indicated that AI/AN/NH college students who experienced problems in their social bonds (i.e., experience loneliness, family problems, difficult social relationships, and intimate partner violence) were more likely to misuse opioids, Qeadan et al.’s [14] study underscored the importance of healthy social relationships as a protective factor against opioid use among AI/AN/NH college students.

With a social support construct created by combining answers to eight survey questions covering four domains, including family, school, community, and peers, Agyemang et al.’s [20] study found that AI/AN high-school students who reported having high social support were less likely to misuse opioids.

In West et al.’s [13] qualitative study, the theme of resiliency was identified as a protective factor that reduced the likelihood of AI/AN youth developing substance and opioid use disorders. Resilience was described in terms of family cohesion, cultural practices/cohesion, family and community support. Participants specifically mentioned family in terms of a strong sense of togetherness, participation in cultural/traditional practices that promote a sense of community, and receiving community and family support in times of need as protective factors against engaging in opioid or substance use. Similar views were expressed in Zeledon et al.’s [28] study. Participants, who were all healthcare professionals, pointed to cultural connectedness/cohesion (i.e., the extent family units and community members participate in cultural activities) and family dynamic (i.e., immediate and extended family supporting, caring for, and nurturing one another) as opportunities that help AI/AN individuals feel connected and protected against opioid and substance use (see Table 4).

The studies included in the review identified other non-social factors that served as risk and protective factors for opioid use or misuse among AI/AN populations. In Prince et al.’s [27] study, the machine learning algorithm identified reporting recent cocaine use, having ever tried a narcotic other than heroin, and identifying as American Indian as the three most salient risk factors of opioid use among American Indian youth. In this same study, it was found that initiating substance use at a later age served as a protective factor against past-30-day opioid use [27]. West et al. and Zeledon et al. [13,28] noted mental health comorbidities (specifically, anxiety, depression, and PTSD) as issues that lead one to use or misuse opioids and abuse other substances. Agyemang et al. [20] and Qeadan et al. [14] also found in their studies that AI/AN students with poor academic performance (e.g., making C, D, or F grades) were more likely to misuse opioids. In both studies, however, it was found that being old and feeling safe on campus were associated with a decrease in the odds of misusing non-prescription opioids [14], and higher maternal education served as protective factor against opioid misuse among middle- and high-school AI/AN students [20].

### 3.5. Addressing Opioid Use/Misuse

Although the general focus of the studies included in this review was on understanding the risk and protective factors of opioid use in AI/AN populations, most of the studies highlighted ways to address the problem of opioid use or misuse in AI/AN populations.

In Hirchak et al.’s [26] study, the importance of resilience was underscored by the finding that increased levels of social and community support have greater protective effects on not using non-medical prescription opioids among AI/AN youth as they progressed in school. The authors explained how this finding could offer evidence for clinicians and prevention scientists in determining appropriate times for intervention and the centrality of resiliency (social and community support) as an intervention designed to prevent the initiation or the progression of non-medical prescription opioid use among AI/AN youth.

Staying socially engaged is a known protective factor against opioid use [27]. Social media is a proxy for social engagement. With their finding that Snapchat use protects against opioid use among AI youth, Prince et al. [27] noted the significance of social media as a means of building social capital that might help individuals feel connected and reduce their risk of engaging in substances. Prevention and intervention strategies, as they noted, should include factors such as social media engagement that are often overlooked in national conversations around the opioid epidemic.

Qeadan et al. [14] noted in their study that social relationships fraught with problems increase AI/AN/NH college students’ risk of opioid misuse. With the findings underscoring the importance of healthy social bonds as a protective factor against opioid misuse, the authors pointed to the need to consider interpersonal relationships (e.g., peer, family) in opioid use prevention interventions for AI/AN/NH youth and young adults.

West et al. [13] articulated in their study the need for intervention programs to be founded on cultural beliefs and practices. They suggested developing services that enhance resiliency and address family and community factors that increase AI/AN youth risk of opioid use.

## 4. Discussion

The opioid epidemic has significantly impacted American Indian and Alaska Native populations in the United States [29]. Opioid-related overdose death rates for AI/ANs have risen almost continuously for more than two decades [28,29,30], with the largest increase occurring between 2019 and 2021 [29]. Informed by the analysis of seven studies published between 2010 and 2022, the current study took stock of social factors that influence opioid use or misuse among AI/AN populations. By focusing specifically on AI/AN populations, this review is the first to attempt to not only synthesize results from a complex body of literature but also to highlight a racial group that has been disproportionately impacted by the opioid crisis and yet is often left out in opioid-related conversations [30,31]. It is important to note that this review excluded studies published after 2022 to minimize the confounding influence of COVID-19 on opioid use and misuse patterns; however, future updates should incorporate post-2022 literature to examine potential pandemic-related shifts in trends of opioid use or misuse among AI/AN populations.

The measure of opioid use or misuse in the studies included in this review varied, from 30-day/past-month use to 12-month use, and lifetime use to simply opioid use. While different figures were reported, giving indication of the prevalence of opioid use or misuse in the study sample, one thing was clear: opioid use continues to be major problem impacting AI/AN populations. Although indigenous populations are known to be impacted by the opioid epidemic at a disproportional rate [30,31], the fact that the majority of the studies included in this review focused on AI/AN youth, particularly students (from middle school to college), illustrates the pervasiveness of this issue and demonstrates the need for age-appropriate interventions to address this growing public health concern. Research suggests that substance use is an important contributor to many mental health and behavioral issues, social ills, and economic and legal problems, and it typically is initiated during adolescence [32,33,34]. For indigenous youth, however, the rate of their experience of these issues, particularly mental and behavioral problems, is 2.5 times higher than the national average [35]. What is not clear from five of the studies included in this review is whether opioid use in the samples studied is the result of historical and intergenerational trauma. It is important to note, however, that in Prince et al.’s [27] study, identifying as AI was the third most salient predictor of opioid use.

Although not operationalized as a variable that could be measured, historical and intergenerational trauma were pointed out by participants in West et al. [13] and Zeledon et al.’s [28] studies as factors that increase AI/AN populations’ risk of opioid use or misuse. Historical trauma has been described as emotional and psychological harm experienced over time and across generations by a group of people who share an identity or affiliation [36,37,38]. Historical trauma has been found to be associated with poor health outcomes in AI/AN populations [36,39,40]. Even though its connection to poor health outcomes remains a subject of debate, as studies have reported mixed results, with no coherent conclusion about the association [41], research shows that individuals with a history of trauma use more substances and are at higher risk for dependence, substance use disorders, and related complications [42,43,44].

Historical trauma does not just refer to events that occurred in the past. It also refers to contemporary challenges, through awareness of historical loss and microaggressions—everyday experiences of racism, discrimination, and hassles targeted at AI/AN individuals [37]. Historical trauma transmitted intergenerationally diminishes family functioning and impairs parenting practices [37,45]. Faced with these challenges, young AI/AN individuals often turn to substances to cope [37,46].

In line with the objective of this review, the studies included in this research identified a number of social factors that increase AI/AN populations’ risk of opioid use or misuse. The studies specifically identified family problems [13,14,28], difficult social relationships, intimate partner violence, loneliness [14], family substance use and low family disapproval of substances [13,15,28], peer influence [15,27], low social support [20], community normalization of substance use, community stressors, disconnection from community and culture, and historical and intergenerational trauma [13,28] as social factors which increase AI/AN (and NH) populations’ risk of opioid use or misuse. These are consistent with the findings of studies by Jalali et al. [47], Jones et al. [48], Stumbo et al. [49], and Keyes et al. [50], who identified interpersonal relationships (e.g., family with OUDs, family history of opioid use, parental disapproval of substance use, friend/co-worker use of opioids, etc.) and conditions of community and society (e.g., community normalization of opioids, easy access to legal and illegal opioids, etc.) as social factors that put people at risk of using or misusing opioids.

Similarly, the studies included in this review also identified different social factors that serve as protective factors against the use or misuse of opioids in AI/AN populations. Social factors that were identified as protective for AI/AN populations against opioid use or misuse included family and community support [13,26,28], family cohesion and cultural connectedness [13,28], clear rules on substance use at school and better school performance [15,26], family disapproval of opioid use, the use of Snapchat, less pressure from friends to use drugs, having greater levels of social support [26,27], and healthy social bonds [14]. These findings are consistent with the work of Zuckerman et al. [51] who found elements in a person’s life that make it easier to avoid the use or misuse of opioids. For instance, Zuckerman et al. [51] found being strongly connected to school and receiving adult support prevented youth from non-medical use of prescription opioids. With their potential to devalue any actions that can cause harmful health effects, such as substance use, Dickerson et al. [52] and D’Amico et al. [53] noted that having healthy social networks and social support, being strongly connected to culture, and actively engaging in cultural practices and activities could protect AI/AN populations, particularly emerging adults, from opioids by helping them navigate a sober and healthy life.

Altogether, social network factors such as family, community, peers, education, culture and tradition, and social media play vital roles in opioid use or misuse among AI/AN populations. Judging from the number of studies included in this review, however, it is reasonable to conclude that little attention has been paid to the opioid problem as it relates to AI/AN populations, both scholarly and legislatively [30]. The opioid crisis has been widely studied in the general population, with multiple preventive and treatment efforts undertaken to address the issues. The same, however, cannot be said about AI/AN populations that continue to experience higher rates of opioid-related deaths compared to other ethnic groups [28]. Having documented some of the social factors that influence opioid use, attention needs to shift to ways to effectively address opioid use or misuse in the AI/AN populations. Although the focus of most of the studies included in this review was not on addressing the opioid problem in AI/AN populations, these studies hinted at what is important to consider when contemplating what preventive and/or treatment interventions to adopt.

Addressing the opioid crisis among AI/AN populations requires understanding its historical roots, including punitive policies from the 1970s that targeted minority communities [54]. These approaches, focused on punishment rather than support, continue to negatively impact current efforts, particularly in AI/AN communities that depend on cultural and community ties. While recent national strategies, like the 2019 roadmap from the White House Office of Science and Technology Policy [55], have begun recognizing the need for group-specific responses, they often lack a full understanding of how the crisis is embedded in broader systems like healthcare and criminal justice [30]. More promising are initiatives like the National Institute of Health’s Helping to End Addiction Long-Term (HEAL) Prevention Initiative, which incorporates culturally relevant strategies, community involvement, and institutional collaboration tailored for AI/AN populations [56]. This is echoed in some of the studies included in this review which note that efforts, including policy interventions, to address the opioid problem require an integrated approach that is evidence-based, socially informed, and grounded in culturally relevant content, necessary to building resilience in AI/AN individuals and communities [13,28].

## 5. Limitations of Study

The current study set out to review primary studies, written in English, published between 2010 and 2022, focused on AI/AN populations in the United States, and accessed through three scholarly databases, five institutional repositories (scholar(ly) commons), and one academic search engine (Google Scholar). Although the opioid epidemic seems localized, disproportionately affecting indigenous populations in the United States, it is important to note that there are similar native populations elsewhere in the world (e.g., Canada and Australia) that might be dealing with similar opioid problems. While limiting this review to studies conducted in the United States may have excluded other valuable studies conducted on similar native populations elsewhere, the decision to focus on AI/AN populations in the United States was informed by the need to maintain methodological consistency and relevance, as well as the unique historical, legal, cultural, and policy environment that shapes the experiences and health outcomes of AI/AN populations in the United States. The information contained in this review is also limited to the extent of information obtained from the available primary studies.

## 6. Future Directions and Implications

The current study highlights different social–ecological factors that influence opioid use among AI/AN populations. The evidence largely points to the important role that family, peers, community, culture, education, and social media play to either protect AI/AN populations or put them at risk of opioid use. A significant proportion of the participants included in the primary studies were adolescents and young adults, giving an indication that these groups are susceptible to opioid use. This presents an opportunity for early intervention that might change the trajectory of the opioid crisis as it relates to AI/AN populations. The path to move forward must include interventions and initiatives that capitalize on AI/AN populations’ strengths and resilience and prioritize the needs of AI/AN youth.

Problems pertaining to social bonds appear to play a significant role in opioid use. It is recommended that future programs develop early intervention strategies for AI/AN youth and young adults that seek to address social relationship risk factors. Such interventions should also incorporate cultural traditions and practices known to enhance resilience among AI/AN populations and are protective against opioid use. On a legislative level, more resources should be allocated to AI/AN populations to aid in the development of family-, peer-, and school-level interventions to address disparities in opioid use and consequences.

The finding that self-identifying as AI/AN increases a person’s risk of opioid use warrants further investigation. While the association between historical/intergenerational trauma and substance use has been the subject of research, it is important to note that results of studies have been inconclusive [41]. The studies included in this review did not establish any relationship between identifying as AI, historical/intergenerational trauma, and opioid use. Longitudinal studies are needed to confidently establish the link between historical/intergenerational trauma and opioid use, as many of the studies available are cross-sectional where the direction of causality cannot be determined.

Social network analysis studies examining social relationships and opioid use are needed to understand the structure, patterns, and implications of social relationships and how they may impact opioid use among AI/AN populations. Such studies could also explore and provide insight into how social networks can be used to identify, access, and engage high-risk, difficult-to-reach AI/AN populations for prevention and treatment.

Finally, the important role that social media plays in opioid use or misuse needs further examination. Future research should investigate and shed more light on risk versus protective engagement in social media with respect to opioid use among AI/AN populations.

## 7. Conclusions

This study illuminated the state of research on social network domains and factors that influence opioid use in native populations. A key takeaway was that study samples involved mostly middle- and high-school as well as college students which, to some degree, shifts the perspective regarding the opioid problem, as it affects native populations and helps structure more targeted interventions to address this issue. The study highlighted the fact that family, community, peers, education, culture, and tradition play vital roles in opioid use or misuse among AI/AN populations. Scholarship in this area can be strengthened by the inclusion of network analysis research that examines structure and patterns of behavior and interactions of various elements within one’s network that influence opioid use or misuse. Based on the findings of this systematic review, increased resources must be devoted to addressing the opioid problem that has for a lengthy period of time disproportionately impacted AI/AN individuals and communities. The findings demonstrate a need for preventive interventions that are evidence-based, incorporate attention to family, peer, and community risk and protective factors, and are grounded in culturally relevant content, necessary to building resilience in AI/AN individuals and communities.

## Figures and Tables

**Table 1:** Literature search terms.

Terms Chosen and Combined in the Search					
Database and Repositories	Population		Problem/Outcome		Independent Variable (Risk/Protective Factor)
ProQuest, PubMed, EBSCOhost (academic search elite, health source nursing, APA PsychArticle, APA PsycInfo, Social Science full text, Eric, Academic Search Premier), Google Scholar, Indigenous Studies Portal (I-Portal) scholar(ly) commons ^+^ (UND, ASU, UofU, OU, SC)	“Native Americans” OR “American Indian” OR “Alaska Native” OR “Indigenous People” OR “Native People”	AND	“Opioid use” OR “Opioid misuse” “Opioid Use Disorder” OR “Opioid addiction”	AND	“Social support” OR “Social support network” OR “Social network” OR “Support network” OR “Social network domains” OR “Social relation *” OR “Familial relation *” OR “Peer relation *”

Notes: *—used in literature search to find alternate endings for search terms (e.g., social relation * = social relations/relationship/relationships). Scholarly common ^+^: UND—University of North Dakota; ASU—Arizona State University; UofU—University of Utah, OU—University of Oklahoma; SC—University of South Carolina.

**Table 2:** Inclusion and exclusion criteria.

Variable	Inclusion Criteria	Exclusion Criteria
Problem/outcome	Opioid use/misuse/opioid use disorder/opioid addiction	Substance abuse without the mention of opioid
Risk/protective factor	Social network type as risk and protective factors family, peer, school, community	Does not include any social network measure
Population/location	American Indian, Native Americans, Alaska Natives United States	Outside the United States
Study design	Qualitative/Quantitative	Study protocol/proposed study, editorial, systematic review/scoping review
Publication years	2010–2022	--

**Table 3:** Methodological characteristics of included studies.

Authors and Year of Publication	Study Type/ Design (Data Source)	Sample Size/ Sampling Strategy	Population Type	Measures/ Variables of Interest/Risk or Protective Factor	Opioid Use Measure	Statistical Analysis
Hirchak et al. (2021) [26]	Quantitative Cross-sectional (NM-YRRS, 2013, 2015, 2017)	42,098 (7307 self-identified AI/AN) Stratified subsampling	School-based Middle- and high-school students in rural and urban locations (Grades 8–12)	Resiliency measured by relationships at home (parent/adult), at school (teacher/friend), and engagement in community (clubs, sports team, church)	Recency: lifetime use of non-medical prescription (middle and high school) Past-30-day use (high school) Method: self-report Type: codeine, Vicodin, OxyContin, hydrocodone, Percocet	Logistic regression
Prince et al. (2021) [27]	Quantitative Cross-sectional (OYOF, 2015–2017)	6482 (3098, self-identified as AI, including those who identified as AI and another race) Convenience sampling	School-based Middle- and high-school students (Grades 7–12)	Cultural and religious identity, family structure and values, peer norms and influence, and Snapchat use	Recency: past-30-day use of opioid Method: self-report Type: cocaine, heroin, Demerol, Vicodin, OxyContin, opium Other substances: alcohol, cannabis, cocaine, amphetamines, huffing glue	Recursive partitioning, a type of decision tree-based machine learning
Nalven et al. (2020) [15]	Quantitative Cross-sectional (ADAS—CSU TEPR, 2009–2013)	5774 (3498 self-identified AI) Stratified subsampling	School-based Middle- and high-school students (youth) living on or near reservations (Grades 7–12)	Family measures (family caring, disapproval of substance), peer substance use, and school measures (school performance, attitude toward school)	Recency: past-month misuse of opioids, lifetime use of opioids, past-month heroin use, lifetime heroin use Method: self-report Type: heroin, codeine, methadone, morphine, OxyContin, opium Other substances *	Multilevel logistic regression
Qeadan et al. (2021) [14]	Quantitative Cross-sectional (ACHA-NCHA II)	8094 AI/AN/NH Stratified subsampling	School-based College students	Social relationship problems (loneliness, difficult social relationships, family problems, intimate partner violence)	Recency: prescription opioid misuse and non-prescription opioid use within the last 30 days and within the last 12 months Method: self-report Type: opiates (heroin, smack), painkillers (OxyContin, Vicodin, codeine)	Multivariable logistic regression Quasi-Poisson regression
Agyemang et al. (2022) [20]	Quantitative Cross-sectional (NM-YRRS, 2009–2019)	3641 AI/AN Cluster	School-based High-school students (Grades 9–12)	Social Support assessed in 4 domains, family, school, community, and peers, with questions on parental interest in school work, teacher’s belief in student’s success, and adult and friend in community who cares about the student	Recency: lifetime use of heroin and past 30 days use of painkillers Method: self-report Type: Heroin, Vicodin, OxyContin, Percocet	Linear Regression
West et al. (2021) [13]	Qualitative (California Opioid Needs Assessment)	83 AI/AN youth Participants (ages 13–18 years) Convenience	Community-based (AI/AN communities in California)	Community substance use description (e.g., access, family substance use, perceptions of people with OUD/SUD), resiliency/protective factor (e.g., community strength—family cohesion, cultural cohesion—traditional practices), and risk factors (e.g., family stressor, peer substance use, community stressors)	Recency: opioid and other substances use Method: self-report Type: opioids (e.g., codeine, promethazine) and other substances (e.g., cocaine, amphetamines/methamphetamines, Xanax, vaping, prescribed painkillers)	Thematic analysis using NVivo
Zeledon et al. (2020) [28]	Qualitative PAR (California Tribal and Urban Opioid Needs Assessment)	21 (14 self-identified AI health experts) Snowball	Community/service based Healthcare professionals/services providers	Description of substance use in community (e.g., most commonly seen AI communities), risk factors (stressful event that may contribute to opioid use), and protective factors (e.g., cultural cohesion, family dynamics)	Recency: none Method: healthcare professionals note/report Type: commonly identified substances impacting AI/AN communities: opioids, heroin Other substances: alcohol, methamphetamines, marijuana	Thematic analysis using NVivo

Notes: Other substances *—Nalven et al. list no specific opioids used by study participants.

**Table 4:** Study results.

Author(s)	Aim/Purpose of Study	Results
Hirchak et al. (2021) [26]	Investigated the prevalence of non-medical prescription opioid use and factors associated with resilience among AI/AN and non-AI/AN students residing in urban and rural areas, utilizing data drawn from 2013, 2015, and 2016 administration of NM YRRS	No significant differences in non-medical prescription opioid use based on rural versus urban residence among rural and urban students across all three survey years. Similarly, no significant differences in opioid misuse were observed between AI/AN and non-AI/An students. Across all time points, protective factors associated with reduced misuse included strong family and community support, the presence of caring adults, meaningful community engagement, and clear behavioral expectations within schools.
Prince et al. (2021) [27]	Employed a machine learning (ML) approach to examine key risk and protective factors associated with past-30-day opioid use among youth living on or near American Indian reservations	The ML algorithm identified 11 significant predictors. The strongest risk factors included recent cocaine use, prior use of narcotics other than heroin, and AI racial identification. Protective factors included abstention from opioids other than heroin, lower frequency of binge drinking, fewer peers exerting pressure to use illicit substances, later initiation of alcohol use, and older age.
Nalven et al. (2020) [15]	Investigated the influence of peer, familial, and school-related factors on opioid use among AI youth	Lifetime opioid misuse was positively associated with higher levels of peer substance use (OR = 1.14, *p* < 0.001), lower perceived family disapproval (OR = 0.98, *p* = 0.01), and poorer academic performance (OR = 0.90, *p* = 0.01). Past-month opioid misuse was similarly associated with increased peer substance use (OR = 1.05, *p* < 0.001), and reduced family disapproval (OR = 0.99, *p* = 0.04). Among all variables, peer substance use emerged as the only significant predictor of both lifetime (OR = 1.15, *p* < 0.001) and past-month heroin use (OR = 1.02, *p* = 0.047).
Qeadan et al. (2021) [14]	Aimed to estimate the prevalence of opioid misuse among AI/AN/NH college students and assess whether disruptions in social bonds contribute to elevated misuse of opioid within this population	Opioid misuse was most prevalent among AI/AH/NH students (7.12%) compared to students from other racial and ethnic groups. AI/AN/NH students reporting experiences of loneliness (aOR = 1.68), strained social relationships (aOR = 1.27), family difficulties (aOR = 1.32), and intimate partner violence (aOR = 1.92) demonstrated significantly higher odds of opioid misuse relative to peers not reporting such challenges.
Agyemang et al. (2022) [20]	Analyzed longitudinal trends in opioid misuse, social support, and suicide attempts among AI/AN high-school students in New Mexico between 2009 and 2019	Prevalence of suicide attempts remained statistically unchanged over the study period but was consistently higher among female students, those reporting opioid misuse, low social support, low maternal education, poor academic performance (grades C or below), and those identifying as non-heterosexual. In 2009, AI/AN students who reported opioid misuse were significantly more likely to have attempted suicide compared to their peers who did not misuse opioids (35.8% vs. 10.4%). A statistically significant decline in opioid misuse was observed from 2009 to 2017 (16.1% to 8.8%, *p* = 0.0033), with similar patterns across sex (males: 15.9 to 9.0%, *p* = 0.002; females: 16.2% to 8.6%, *p* = 0.012), and among youth with mothers who attained at least a high-school education (13.5% to 6.7%, *p* = 0.019). However, a significant increase in opioid misuse occurred between 2017 and 2019 (8.8.% to 12.9%, *p* < 0.0001). In 2019, students with low social support had markedly higher rates of opioid misuse (18.9% vs. 8.5%, *p* < 0.0001) and suicide attempts (21.3% vs. 7.0%, *p* < 0.0001), compared with those with high social support.
West et al. (2021) [13]	Examined AI/AN youth experiences with opioid and other substances use disorders in AI/AN communities	The theme of risk factors had participants identifying mental health, community stressors, peer pressure/social norms, family substance use, and family stressors as important attributes that contribute to opioid use/misuse. Themes identified pertaining to resiliency or protective factors included family cohesion and community support, adherence to cultural/traditional practices, and the development of culturally based youth programs.
Zeledon et al. (2020) [28]	Identify facilitators and barriers to treatment of substance use disorders (SUDs) and opioid use disorders (OUDs).	Themes identified pertaining to risk factors were the effects of historical and intergenerational trauma on wellbeing of community members and family units; disconnection from community or events that interfere with the individual’s sense of belonging to their community; mental health comorbidities such as anxiety and depression; polysubstance use; normalization of OUD; and economic stress. Themes identified pertaining to protective factors were cultural cohesion, described as “the extent to which family units and community members participate in cultural activities”, and family dynamics, described as “immediate and extended family supporting, caring for, and nurturing one another.” Both themes were presented as offering opportunities to feel connected and have a sense of belonging that could protect individuals from OUDs.

## Abbreviations

The following abbreviations are used in this manuscript:

AI/AN/NH	American Indian and Alaska Native/Native Hawaiian
OUD	Opioid Use Disorder
SUD	Substance Use Disorder
PRISMA	Preferred Reporting Items for Systematic Reviews and Meta-Analyses
NM-YRRS	New Mexico Youth Risk and Resiliency Survey
OYOF	Our Youth, Our Future Survey
ADA	American Drug and Alcohol Survey
ACHA-NCHA	American College Health Association National College Health Assessment II
